# Raccoon roundworm prevalence (*Baylisascaris procyonis*) at the North Carolina Zoo, USA

**DOI:** 10.7717/peerj.9426

**Published:** 2020-07-20

**Authors:** Meghan M. Louis, Larry J. Minter, James R. Flowers, Michael K. Stoskopf, Suzanne Kennedy-Stoskopf

**Affiliations:** 1 Department of Clinical Sciences, North Carolina State University College of Veterinary Medicine, Raleigh, NC, United States of America; 2 Environmental Medicine Consortium, North Carolina State University, Raleigh, NC, United States of America; 3 Hanes Veterinary Medical Center, North Carolina Zoo, Asheboro, NC, United States of America; 4 Department of Population Health and Pathobiology, North Carolina State University College of Veterinary Medicine, Raleigh, NC, United States of America

**Keywords:** Raccoon, Procyon lotor, *Baylisascaris procyonis*, Raccoon roundworm

## Abstract

*Baylisascaris procyonis* is an important zoonotic nematode of raccoons (*Procyon lotor*). Infection with this parasite has important health implications for humans, zoo animals, and free-ranging wildlife. As a large, natural habitat zoo, the North Carolina Zoo (NC Zoo) coexists with native wildlife. Raccoons are abundant at the NC Zoo and the prevalence of *B. procyonis* is unknown. Raccoon latrines were located through employee reporting and systematic searching throughout the zoo and sampled for *B. procyonis* in October and November of 2018 and 2019. Parasite prevalence, latrine location, substrate category and latrine persistence were recorded. Thirty-three latrines were located in 2018 and eight new latrines in 2019 while four latrines from the prior year were no longer available to be sampled. Of the 29 latrines sampled over the two years, 16 (55%) persisted for at least one year. The majority of the latrines were found on natural substrate with rock showing the highest preference. Just over half (n = 21 of 41 total) of the active latrines in the study were in or immediately adjacent to animal enclosures. Two latrines were found in public areas including one contaminating children’s play equipment. Additionally, fresh fecal samples were collected from five adult raccoons presented to the zoo’s veterinary clinic in 2018 and 2019. All fecal samples tested by centrifugal flotation for both years were negative for *B. procyonis*. The results of this study show the value of field sampling to properly assess risk and enable informed decision-making regarding public health and wildlife management.

## Introduction

*Baylisascaris procyonis* is a zoonotic parasite found in raccoons (*Procyon lotor*). Raccoons defecate in communal locations called latrines that can serve as important foci for *B. procyonis* transmission because the parasite ova are shed in feces and can persist in the environment for many years (Hirsch et al., 2014). The roundworm, *B. procyonis* does not cause clinical disease in raccoons but can be fatal to humans and many other species. The disease has been well summarized in a recent major review that summarizes the clinical disease observed in over 150 species of mammals and birds (Kazacos, 2016). Neural, visceral, and ocular larva migrans are potential sequelae secondary to infection and aberrant migration of *B. procyonis* in paratenic hosts, including humans (Graeff-Teixeira, Morassutti & Kazacos, 2016).

*Baylisascaris procyonis* was first reported in a zoological setting during the 1930s in raccoons in the New York Zoological Park (McClure, 1933; Kazacos, 2016). Zoological institutions have continued to be at the forefront of knowledge and discovery regarding *B. procyonis* (Kazacos, Fitzgerald & Reed, 1991; Campbell et al., 1997; Sexsmith et al., 2009; Zimmerman, Dangoudoubiyam & Kazacos, 2019). The North Carolina Zoo (NC Zoo) is situated on 1,050 wooded hectares in Randolph County, North Carolina, USA (https://www.nczoo.org/visit/about-zoo). Raccoons likely have found the zoo a hospitable location to thrive. There is a healthy, forested landscape in addition to access to food and water resources within animal exhibits and refuse deposited by visitors in garbage receptacles. The NC Zoo does not mitigate for the presence of raccoons so little to no anthropogenic mortalities occur on the grounds.

*Baylisascaris procyonis* was historically thought to be absent from North Carolina and much of the southeastern United States. Helminth surveys from five Southeastern states over a 32-year period did not find *B. procyonis* in raccoons (Harkema & Miller, 1964; Miller, 1992). The parasite was detected for the first time in North Carolina in 2013 along the western border with Tennessee (Hernandez et al., 2013). Reports in the southeastern region of the United States suggest that this parasite is either spreading to new regions or has been present in unrecognized locales (Blizzard et al., 2010).

The objective of this study was to ascertain the prevalence of *B. procyonis* at the NC Zoo to assess the risk to collection animals, humans, and native wildlife. The results of this study will guide future raccoon and latrine management decisions at the NC Zoo.

## Materials & Methods

The study occurred within the 200 developed hectares of the zoo, where raccoon-human-zoo species interactions were considered most likely. Raccoon latrines were identified based on fecal characteristics and contents (Yeager & Rennels, 1943), and coordinates were recorded using a Global Position System application (Motion X GPSTM, Fullpower Technologies, Inc, Santa Cruz, CA, USA). Employee reporting combined with systematic searching of zoo grounds and enclosures were used to locate latrines. Systematic searching was accomplished by one to two people walking line transects 300 meter long and 20 meter wide in regions void of enclosures and fence barriers. Setting and searching transects took approximately 45 min if walked by two people and double that when searched by a single person.

Within enclosures, both natural features and man-made structures were targeted based on known raccoon preferences for elevated horizontal surfaces (Yeager & Rennels, 1943; Stains, 1956; Armstrong et al., 1989; Cooney, 1989; Kazacos & Boyce, 1989; Page, Swihart & Kazacos, 1998). Latrines were then categorized based on the underlying substrate. Natural environment categories included rock, ground, and wood (stumps and logs). The man-made environment was separated into cement (walls, flooring, and enclosure structures) and wooden structures (barn, roof, and decks).

Fecal piles less than 2 m apart were considered from the same latrine. Identified latrines were sampled during the months of October and November of 2018. These latrines were revisited and sampled in October and November of 2019, and systematic searches conducted to identify additional latrines. Sampling of latrines occurred in the fall, when the highest prevalence of *B. procyonis* has been documented in raccoon feces (Kidder et al., 1989; Page et al., 2016).

The freshest one to three scats were collected from each latrine, placed individually in a sample container, and processed the same day. Individual scats were collected rather than pooling samples to decrease bias regarding latrine size characteristics (Smyser, Page & Rhodes, 2010). The freshest feces were collected to minimize the risk to personnel of larvated ova, the infectious stage of the parasite, which develop anywhere from two to four weeks post-defecation. Fresh feces were also collected from five adult raccoons found on zoo grounds that presented to the zoo veterinary hospital at various times during 2018 and 2019.

Approximately 2 grams of feces from each fecal sample were mixed with Sheather’s sugar (specific gravity of 1.27) solution (Jorgensen Laboratories, Loveland, CO 80538, USA), centrifuged and microscopically examined for the presence of *Baylisascaris* ova as per Dryden et al. (2005). Samples that were chosen with no particular pattern were submitted for confirmatory examination by a veterinary parasitologist at North Carolina State University College of Veterinary Medicine (NCSU-CVM). The presence of *B. procyonis* or lack thereof, was based on size and morphologic appearance of ova observed in the flotation mounts.

## Results

Sixty-two individual fecal samples from 33 raccoon latrines were examined in 2018. All samples were negative for *B. procyonis*. Twenty-nine of the latrine sites were revisited in 2019 and 16 latrines (55%) were still active, meaning fresh feces had been deposited. Four latrine sites from 2018 could no longer be accessed. Eight new latrines, however, were identified. Forty-four individual fecal samples were collected from the 24 identified latrines in 2019 and all samples were negative for *B. procyonis*. Fecal samples representing 25 of the 41 total latrines (61%) that were examined at NCSU-CVM were also confirmed negative for *B. procyonis* ova. The five fresh fecal samples from raccoons that presented to the zoo veterinary hospital during the study period were negative for *B. procyonis*. Evidence of other parasites was noted, including strongyle and capillaria ova, and coccidia and monocystis oocytes. No counts and definitive identifications were made.

**Figure 1:**
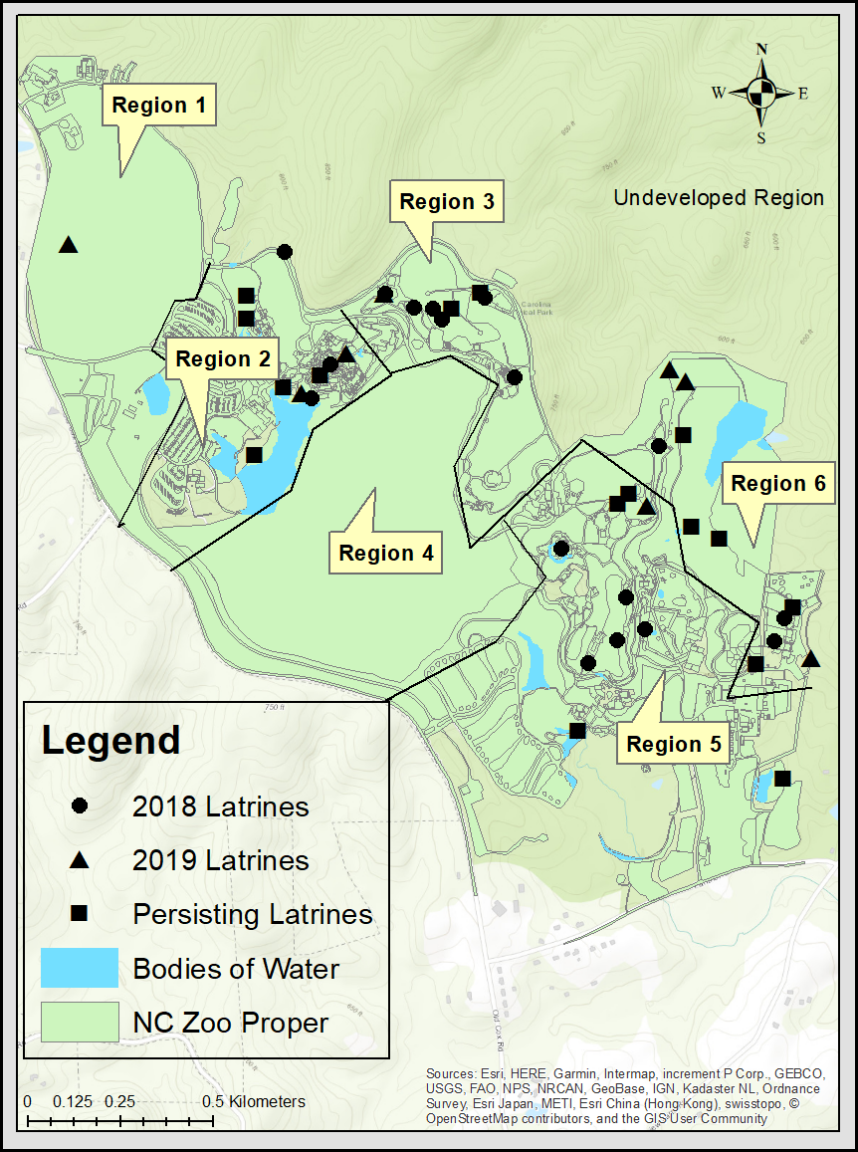
A map of the North Carolina Zoo. The map displays raccoon latrines located in October and November of 2018 and 2019, as well as highlights latrines persisting between the years. The zoo proper is divided into 6 regions with region 2, 3, 5, and 6 being more developed. Sources: Esri, HERE, Garmin, Intermap, increment P Corp., GEBCO, USGS, FAO, NPS, NRCAN, GeoBase, IGN, Kadaster NL, Ordnance Survey, Esri Japan, METI, Esri China (Hong Kong), swisstopo, ©OpenStreetMap contributors, and the GIS User Community.

Of the 41 total latrines sampled between 2018 and 2019, 21 (51%) were in or adjacent to animal enclosures, while 20 (49%) were outside of animal areas (Fig. 1). Two latrines (5%) were present in public areas, whereas the rest of the latrines 39 (95%) were in locations only accessible to zoo personnel (including animal habitats).

Seventeen of the 41 (41%) latrines were located on man-made structures, whereas the remaining 24 (59%) were located on natural substrate. Of those 24, a further division into rock, ground, and wood accounted for 12, 7, and 5 latrines, respectively. Of 17 latrines on man-made structures, 6 were on cement and 11 on wooden structures.

## Discussion

*Baylisascaris procyonis* was not detected in fecal samples from latrines at the NC Zoo. Raccoon latrine prevalence surveys are considered the best method for *B. procyonis* surveillance because they provide a direct assessment of the risk of transmission of *B. procyonis* to humans and animals (Page et al., 2005; Sexsmith et al., 2009; Smyser, Page & Rhodes, 2010). There is a possibility of not detecting a very low prevalence, and this would be expected to be no more than 3% based on our sample size (Wobeser, 2007). The parasite has not been found previously on routine fecal examinations or necropsy of collection animals at the zoo, despite awareness that it is a potential problem. Based on our results, we believe that *B. procyonis* is not currently present at the NC Zoo.

*Baylisascaris procyonis* eggs are hardy and have been shown to persist in the environment for years (Kazacos, 2016; Ogdee, Henke & Fedynich, 2016). A female worm can produce an estimated 115,000–179,000 eggs/day, with the potential to increase to millions of eggs/day with increased worm burdens (Snyder & Fitzgerald, 1987). Sampling of latrines occurred in October and November to coincide with juvenile raccoon dispersal when young raccoons are more likely to shed ova than adults during this period (Prange, Gehrt & Wiggers, 2003; Jardine et al., 2014; French et al., 2019). Although, Kresta, Henke & Pence (2010) found that adult raccoons were just as likely to have *B. procyonis* if the parasite is new to the region.

Our sampling occurred over two years to provide information on latrine persistence, which is important regarding aspects of environmental contamination. Approximately half of the latrines persisted through the study (Fig. 1), although four of the 33 latrine sites could not be revisited during the second sampling period due to either enclosure modifications or animal management changes that precluded access. While the results of our study indicated low/no *B. procyonis* prevalence, knowledge of latrine site persistence is important for future surveys and potential modeling. Latrines appeared to be distributed throughout the more developed portions of the park (Fig. 1). Raccoons may select for more developed regions due to resource distribution, however, this was not tested in the scope of this study (Prange, Gehrt & Wiggers, 2004). One latrine was found in region 1, a less developed area of the park. There is a high likelihood that other latrines exist in these regions, however, increased leaf litter, abundant raised tree crotches and rock outcroppings, and forest cover may have decreased our detection ability.

**Figure 2:**
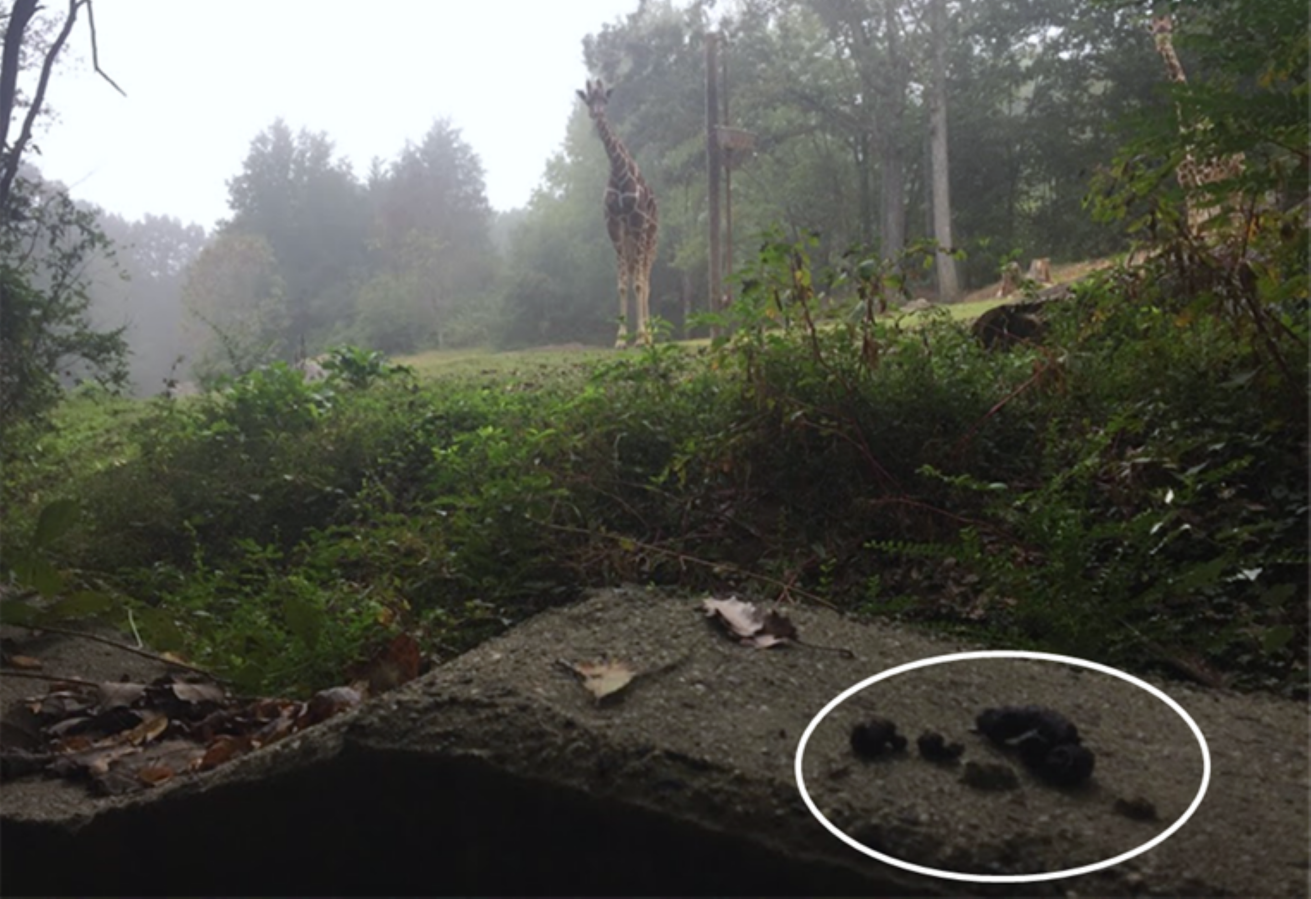
Raccoon latrine on an exhibit wall at the North Carolina Zoo. A raccoon latrine on the man-made cement wall in region 5 at the perimeter of a mixed-species exhibit of zebra (*Equus quagga*), ostrich (*Struthio camelus*), and giraffe (*Giraffa camelopardalis*) at the North Carolina Zoo in the fall of 2018. The white oval is identifying a raccoon latrine on the perimeter wall of the exhibit, which demonstrates the potential exposure of both zoo personnel and exhibit animals. Photo by Meghan Louis.

**Figure 3:**
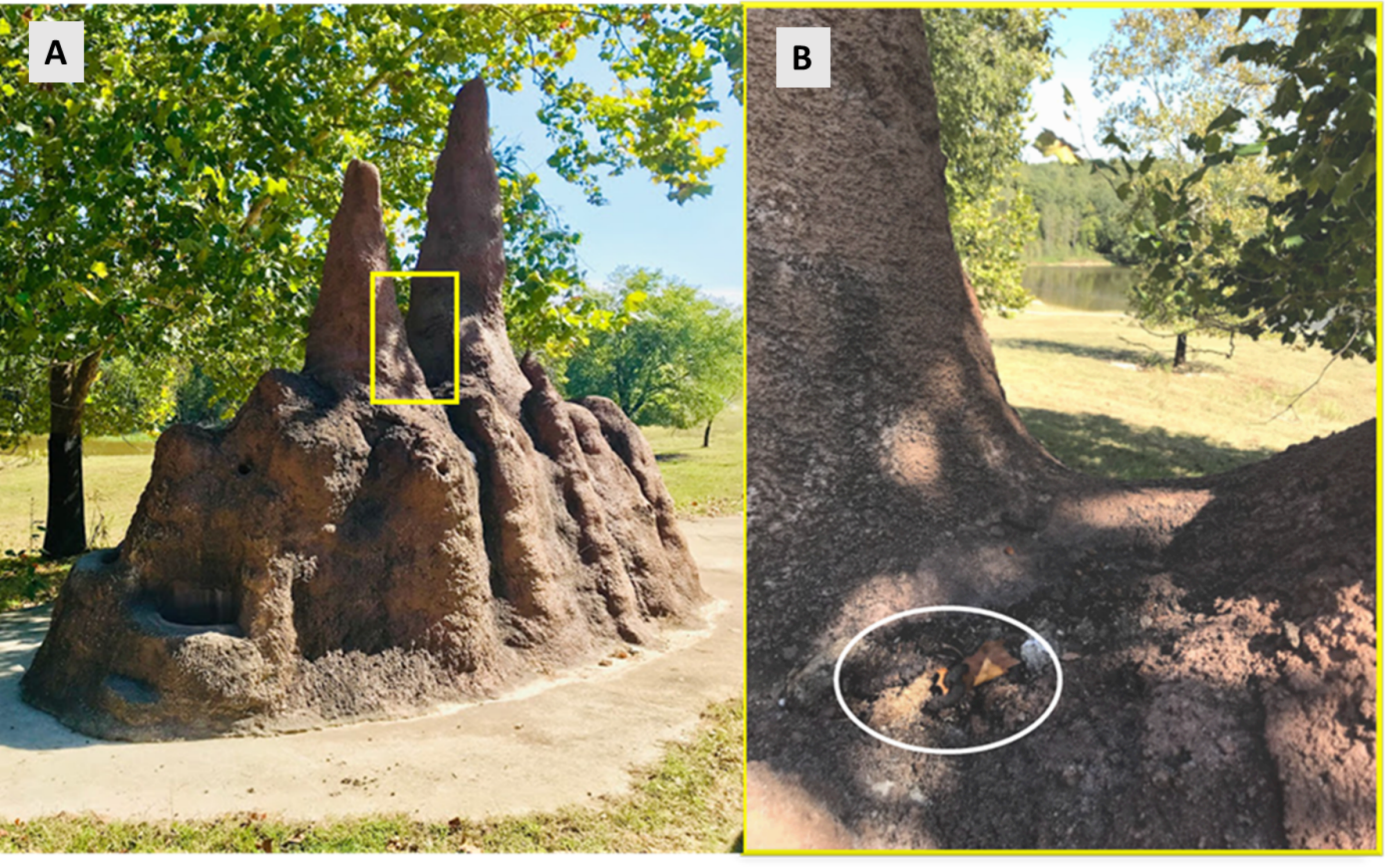
Raccoon latrine on a faux termite mound on the African habitat. A raccoon latrine found in the crux of a man-made termite mound located on a large mixed-species African habitat in region 6 at the North Carolina Zoo in the fall of 2018 and 2019. (A) An overview of the man-made termite mound. (B) Displays a zoomed-in view with a white oval identifying the raccoon latrine. This man-made mound has similar features as a preferred natural substrate of a tree crux. Photo by Meghan Louis.

Raccoon selection of latrine sites shows preference for elevated horizontal surfaces with common locations being on large rocks, logs, at the base and on large limbs of trees and in raised crotches (Yeager & Rennels, 1943; Stains, 1956; Cooney, 1989; Kazacos & Boyce, 1989; Page, Swihart & Kazacos, 1998). In the man-made environment, these horizontal surfaces equate to decks, attics, rooftops, and barn rafters (Kazacos & Boyce, 1989). The NC Zoo has a mixture of both natural habitat and man-made structures (Figs. 2 and 3) within individual enclosures and throughout the park. Most latrines were found on natural substrates and over half occurred on rock surfaces, which is a common feature of the NC Zoo due to limestone outcroppings.

Approximately half of the latrines were found in or adjacent to animal enclosures and habitats, often directly accessible to the collection animal. Considering that many fatal cases of *B. procyonis* larval infections have occurred in animals at zoological institutions, the risk to collection animals is important to assess (Armstrong et al., 1989; Kazacos, Fitzgerald & Reed, 1991; Ball et al., 1998; Sato et al., 2005; Thompson et al., 2008). Most of the habitats at the NC Zoo are open with boundaries of cement walls and/or fence-lines. Raccoons are exceptional climbers and while habitats are meant to keep collection animals in, often the boundaries are not impervious to raccoons. Enclosures are designed to meet the needs of the collection animal, which in-turn can provide shelter and a reliable food source for wild raccoons.

One of the latrines in the public area was discovered on children’s play equipment. Given that children under the age of two are the most impacted clinically by *B. procyonis*, identification of the latrine and further demonstration of the absence of the parasite was particularly important (Graeff-Teixeira, Morassutti & Kazacos, 2016; Kazacos, 2016). The other latrine was located on the park entrance bridge utilized by hundreds of zoo visitors and staff each day. The groundskeepers remarked that they continuously removed the feces at that location, but it always reoccurred. The lack of latrines found in public areas is likely secondary to the continual upkeep of the grounds and manual removal of latrines by zoo personnel, who are considered at higher risk for *B. procyonis* infection (Armstrong et al., 1989; Graeff-Teixeira, Morassutti & Kazacos, 2016).

Zoological institutions can serve as surveillance locales providing information regarding the potential risk of *B. procyonis* to surrounding communities. Seroprevalence surveys of non-human primates from zoos and people working in wildlife rehabilitation facilities, suggest exposure to *B. procyonis* without overt clinical disease occurring (Sapp et al., 2016; Weinstein et al., 2017; Zimmerman, Dangoudoubiyam & Kazacos, 2019). Zoological institutions are the perfect One Health intersection of humans, animals, and their environment and can provide invaluable information regarding diseases of public health importance, such as *B. procyonis*.

## Conclusions

*Baylisascaris procyonis* was not found in the latrines at the NC Zoo. Time and effort used to identify latrine sites and test for *B. procyonis* were manageable for 1–2 people and recommended for zoological institutions as well as other public land areas. Our negative results are important locally, providing insight for future surveillance, monitoring, risk assessments, planning, and wildlife management. In a broader sense, our results contribute knowledge regarding present distribution of this parasite.

##  Supplemental Information

10.7717/peerj.9426/supp-1 Supplemental Information 1 Latrine location at the NC Zoo NE- not examined, NA- not applicable.

## Additional Information and Declarations

### Data Availability

The following information was supplied regarding data availability: Raw data is available as a Supplemental File.
